# Preclinical IBD: One more piece of the puzzle

**DOI:** 10.1002/ueg2.12358

**Published:** 2023-01-17

**Authors:** Manasi Agrawal, Joana Torres

**Affiliations:** ^1^ Division of Gastroenterology Icahn School of Medicine at Mount Sinai New York New York USA; ^2^ Center for Molecular Prediction of Inflammatory Bowel Disease (PREDICT) Department of Clinical Medicine Aalborg University Copenhagen Denmark; ^3^ Division of Gastroenterology Hospital Beatriz Ângelo Loures Portugal; ^4^ Division of Gastroenterology Hospital da Luz Lisboa Portugal; ^5^ Faculdade de Medicina Universidade de Lisboa Lisboa Portugal

Over the recent years, increasing evidence has strengthen the concept that inflammatory bowel disease (IBD) diagnosis is preceded by dysregulated immune pathways, altered intestinal permeability, dysbiosis, and other pathogenic mechanisms, that set the stage for disease to become clinically manifest. Understanding this preclinical period^1^ can help deconvolute IBD pathogenesis, lead to prediction tools, novel therapeutic strategies, and preventive interventions.

The study by Rodrigues‐Lago et al provides new insights into preclinical IBD.^2^ Leveraging the population‐based Basque Colorectal Cancer Screening Programme (individuals aged 50–69, invited to undergo colonoscopy following a positive fecal immunochemical test), the authors originally described incidental IBD diagnosis in 0.35% of this asymptomatic cohort.^2^, ^3^ In the current study, they retrospectively explore healthcare and medication utilization in the years preceding IBD diagnosis; 124 individuals with incidental IBD diagnosed during their screening colonoscopy [current study citation] were age and gender‐matched in a 1:3 ratio to 372 healthy controls (HC), and to 305 IBD patients with symptoms prior to IBD diagnosis. The primary endpoints included outpatient and emergency care visits, hospital admissions, radiological examinations, and antibiotics and corticosteroids prescriptions, 3–5 years before diagnosis (excluding the 12 months preceding diagnosis). Compared to HC, individuals with incidental IBD had higher primary and specialized care, and higher use of corticosteroids. Compared to those with incidental IBD, those with symptoms preceding IBD diagnosis had increased utilization of specialist and emergency care, radiological examinations, and antibiotics and corticosteroids prescriptions.

Access to endoscopic and clinical data is a strength of this study. Indeed, while previous studies have reported on biomarkers of inflammation up to 10 years before IBD diagnosis, it remains unclear when mucosal lesions occur.^4^ And while these data do not resolves this question, they add that mucosal inflammation may exist for an unknown period before IBD diagnosis and are likely to be the final step before symptoms ensue. The study is not without gaps such as the lack of information on the type of specialized care attended, indication for healthcare utilization and matching on limited variables. Further, the median age of this cohort, and thereby comorbid conditions and other variables, may limit the generalizability of these data. However, this data are consistent with other reports on increased healthcare utilization preceding IBD diagnosis.^5^ Together, these studies suggest that nonspecific symptoms may be ongoing for many years before diagnosis. Comparatively, those with symptoms leading up to IBD diagnosis had even higher healthcare utilization, implying that gradual escalation of symptoms (and inflammation) may be the final stage leading to IBD diagnosis. These results are aligned with “ECCO's precision medicine workshop” describing the stages of preclinical IBD, and proposing that mucosal damage is progressive, evolving from microscopic into macroscopic lesions, that may trigger symptoms only after reaching a threshold at which functional and sensory derangements occur.^1^

Better characterizing the period preceding IBD diagnosis has implications towards improving IBD prediction and diagnosis. Certainly, IBD, especially CD, has a long preclinical period. In a recent study from the UK, a greater proportion of individuals reported GI symptoms in the 10 year period preceding IBD diagnosis, and fewer than 50% of those diagnosed with IBD were evaluated within 18 months of symptom onset.^6^ Conversely, altered serum biomarkers and increased intestinal permeability for up to 10 years prior to CD diagnosis likely represent the earliest preclinical period.^4^, ^7^ Simultaneous data on biomarkers and intestinal mucosal changes would be key to understand progression of preclinical IBD, however difficult to obtain, so it remains difficult to align the different stages that may precede disease onset. A better understanding of the preclinical period is key to develop predictive and preventive strategies. In rheumatoid arthritis, for example, many patients pass through a phase of vague joint pain and morning stiffness without clinically apparent synovitis. Better delineation of symptoms associated with progression to RA^8^ has allowed rheumatologists to screen for those at risk for disease, narrowing the group of individuals potentially eligible for disease‐prevention trials.^9^, ^10^ Relatedly, overlap across immune‐mediated diseases, both in epidemiology and underlying pathogenesis, may explain, at least in part, increased healthcare utilization in this cohort.^11^ While fecal calprotectin is a robust screening tool for intestinal inflammation, consideration of extra‐intestinal symptoms (ex: arthralgias, fatigue) in the IBD diagnostic algorithm could lead to earlier diagnosis and intervention, or better characterization of “at‐risk for IBD” individuals. Application of stool and serum‐based biomarkers, and intestinal permeability testing over endoscopy in this context is likely to have additional benefits such as lower invasiveness and costs, and greater outreach at the global level.

In summary, this study has shown that diagnosis of IBD is preceded by increased healthcare utilization medication use, further calling attention to the need to better characterize the period preceding IBD onset, with the potential to predict disease onset and/or earlier diagnosis.

## CONFLICT OF INTEREST

The corresponding author confirms on behalf of all authors that there have been no involvements that might raise the question of bias in the work reported or in the conclusions, implications, or opinions stated. MA reports no conflict of interest. JT has received speaker/advisory board fees from Janssen, Abbvie, Pfizer and BMS, and grants from Abbvie and Janssen.

## FUNDING INFORMATION

MA is supported by the National Institute of Diabetes and Digestive and Kidney Diseases (K23DK129762‐02).

## Data Availability

Research data availability is not applicable.
